# PURSUIT Protocol: Development of a Novel Approach to Managing Youth Physical and Mental Health in Schools

**DOI:** 10.3390/children13020297

**Published:** 2026-02-21

**Authors:** Thea Senger-Carpenter, Jocelyn Zuckerman, Audrey Searles, Cara Poland, Crystal L. Cederna, Sarah Nelson, Mallet R. Reid, Kelly Theaker, Steven J. Pierce, Angela Chia-Chen Chen, Natoshia R. Cunningham

**Affiliations:** 1 Department of Family Medicine, College of Human Medicine, Michigan State University, East Lansing, MI 48824, USA; sengerth@msu.edu (T.S.-C.); zucker32@msu.edu (J.Z.); audlynsea@gmail.com (A.S.); reidmall@msu.edu (M.R.R.); 2 Obstetrics, Gynecology, and Reproductive Biology, College of Human Medicine, Michigan State University, Grand Rapids, MI 49503, USA; 3 Charles Stewart Mott Department of Public Health, College of Human Medicine, Michigan State University, Flint, MI 48502, USA; cedernac@msu.edu; 4 Department of Anesthesiology, Critical Care, and Pain Medicine, Boston Children’s Hospital, Boston, MA 02115, USA; sarah.nelson@childrens.harvard.edu; 5 Department of Psychiatry, Harvard Medical School, Boston, MA 02115, USA; 6 Rockford Public Schools, Rockford, MI 49341, USA; ktheaker@rockfordschools.org; 7 Center for Statistical Training and Consulting, Michigan State University, East Lansing, MI 48824, USA; pierces1@msu.edu; 8 College of Nursing, Michigan State University, East Lansing, MI 48824, USA; chenang6@msu.edu

**Keywords:** pediatric, school, cognitive behavioral, pain management, trauma, substance use

## Abstract

Background/Objectives: Physical and mental health symptoms commonly affecting children are often under-addressed given the limited availability of pediatric behavioral healthcare. Training school providers (e.g., nurses, mental health professionals) to address these concerns is a promising strategy to explore, considering the unique level of accessibility afforded by school settings. While our earlier work augmented school providers’ pain management skills, providers desired more comprehensive training and youth support tools. Our team of interdisciplinary academic researchers and community partners will bridge this gap by developing the PURSUIT (Preventing Use of Substances for the Underserved with Innovative Technology) provider training program and companion online self-management platform for youth and caregivers. This protocol paper describes our planned approach to developing, implementing, and evaluating the PURSUIT program. Methods: We will draw from evidence-based cognitive–behavioral, trauma-focused, and mindfulness protocols to develop a comprehensive provider training program and interactive online self-management platform for youth and caregivers. Content areas will include core cognitive–behavioral strategies and specific skills for pediatric pain management, trauma-focused care, and substance use prevention. Innovative technological approaches, such as live and animated videos, will be used to promote user engagement. Academic and community partners will have roles in material co-development. Outcomes of this project will include the PURSUIT training program and self-management platform feasibility and acceptability (e.g., completion/engagement rates, quantitative/qualitative reports), as well as the impact of the training program on provider knowledge and the impact of the self-management platform on youth/caregiver outcomes. Conclusions: Interdisciplinary collaboration and community engagement will be critical to developing and evaluating a provider training program and youth/caregiver self-management platform.

## 1. Introduction

Physical and mental health symptoms are common among youth and frequently co-occur, increasing risk for impairment and long-term negative health effects [1,2,3]. Common physical symptoms include chronic pain, which is characterized by persistent or recurrent pain (e.g., headache, abdominal, musculoskeletal) that lasts for at least three months and impacts up to 20% of youth worldwide [4]. Recent estimates derived from national samples of U.S. children and adolescents indicate a similarly high prevalence of mental health symptoms, including anxiety (20%), low mood (18%), and trauma sequelae (2.17%) [5,6]. Such data suggest that in an average classroom, multiple youth may be affected by one or more types of symptoms. Indeed, close to half of youth with chronic pain may also experience significant mental health problems [7]. Symptom co-occurrence has been well documented and is associated with increased functional, social, and academic impairment, as well as substance use in adolescence [8,9,10,11] and adulthood [12,13,14]. While effective interventions for physical and mental health symptoms such as cognitive–behavioral strategies exist [15,16,17], access to behavioral healthcare providers is extremely limited, including in the state of Michigan [18,19,20].

Novel solutions are needed to increase access to care for common pediatric symptoms. Given the shortage of behavioral healthcare providers, one potential approach is to enhance the training of trusted professionals already embedded in youths’ lives [21]. Schools and the providers within may offer a uniquely accessible entry point to care. In fact, school nurses are often the first point of contact for youths’ physical complaints, including chronic pain and mental health symptoms [22,23]. Yet, with a few exceptions [24,25], most nurses are inadequately trained in the nonpharmacological (i.e., behavioral) strategies recommended for chronic pain management [26]. Moreover, while school-based mental health professionals are equipped to manage youths’ mental health concerns, the majority are not trained to adapt these skills to physical health challenges (e.g., pain), and many schools do not have any embedded mental health professionals [27,28]. Training school health professionals in evidence-based strategies to support youths’ physical and mental health symptoms is therefore a promising approach to be evaluated for its potential to increase access to care, but methods to disseminate these trainings in feasible and widely adoptable ways remain limited.

Our team previously developed and launched the HELP PAIN training program for school providers, which trained these providers in cognitive–behavioral and mindfulness strategies for managing pediatric pain specifically [29]. While HELP PAIN was deemed feasible and increased provider knowledge, our community partners at the Michigan Association of School Nurses (MASN), as well as state-level partners (including an organization that employs over 400 providers serving youth in Michigan schools), called upon us to expand our training(s) to physical and mental health concerns including trauma-related care and substance use prevention. Our partners also requested more flexible training options that would allow providers to selectively engage with materials most relevant to their practice and educational needs. Simultaneously, the Michigan Health Endowment Fund (MHEF; funder of the HELP PAIN project) issued a call for proposals to develop innovative approaches to substance use prevention that utilized technology to increase accessibility. Thus, in response to aligned community needs and funder priorities, we proposed the PURSUIT (Preventing Use of Substances for the Underserved with Innovative Technology) program, which will be developed as a school provider training program and companion youth/caregiver online self-management platform that broadly address common physical and mental health concerns affecting youth. This protocol paper describes the planned development, implementation, and initial evaluation of the PURSUIT program, detailing the procedures and methodologies that will be used to create and assess the provider training program and youth/caregiver self-management platform.

### 1.1. Study Aims and Hypotheses

We detail the planned protocol for the development, implementation, and evaluation of the PURSUIT program. The outcomes of this project will be assessed within the RE-AIM (Reach, Effectiveness, Adoption, Implementation, and Maintenance) framework, using quantitative and qualitative approaches [30,31]. The RE-AIM framework encompasses both the implementation and effectiveness of the PURSUIT training program and companion self-management platform, recognizing the interdependence of these factors (Table 1). The specific aims of the planned project are listed below.

#### 1.1.1. Aim 1

We aim to evaluate the feasibility, acceptability, and appropriateness of the PURSUIT provider training program and to explore its impact on learning outcomes. Feasibility will be assessed using training completion rates (i.e., the percent of providers who complete at least one post-assessment measure, relative to all those who begin at least one training module). Providers will also complete validated quantitative and qualitative measures of feasibility, acceptability, and appropriateness as per our prior work [32,33]. Impacts on learning outcomes will be explored using changes in pre- and post-training knowledge quiz scores [32].

We hypothesize as primary outcomes that (*H1A*) the PURSUIT training program will be *feasible* as indicated by training completion rates > 75% (based on our HELP PAIN project [34]), as well as (*H1B*) *acceptable* and *appropriate*, as reflected by positive qualitative feedback and average scores > 4 for three validated subscales measuring the feasibility, acceptability, and appropriateness of the program (range 1–5; estimate based on our earlier work [34]). Finally, as secondary outcomes, we hypothesize that (*H1C*) providers will demonstrate increased knowledge of symptom management skills following training compared to baseline.

#### 1.1.2. Aim 2

We will investigate providers’ adoption of PURSUIT skills. Specifically, we will explore the percentage of providers who report using PURSUIT skills on a monthly basis for three months after training, the number of youth with whom providers report using skills, and the average number of skills used per youth. We will also explore the frequency of providers’ monthly PURSUIT-related billing submissions and the percentage of providers who report training another professional in PURSUIT skills over three months.

#### 1.1.3. Aim 3

Separately, we will evaluate as primary outcomes the feasibility, acceptability, and reach of the PURSUIT online self-management platform by measuring youth retention and engagement with the platform from pre- to post- assessment (approximately eight weeks), in line with related work [35]. We hypothesize that the PURSUIT self-management platform will be (*H3A*) *feasible* based on the retention (i.e., completion of at least one post-assessment measure) of >75% among those who complete a baseline assessment [36,37]. We hypothesize that the self-management platform will also be (*H3B*) *acceptable* based on >70% of youth who complete a baseline assessment engaging with the platform (i.e., completing at least one activity within the platform) [36], and youths’ positive qualitative feedback.

Finally, as secondary outcomes we will explore whether engaging with the PURSUIT self-management platform is associated with (*H3C*) improvements in youth mental and physical health symptoms and (*H3D*) caregiver mental health symptoms.

## 2. Methods

### 2.1. Study Design

This two-year, Institutional Review Board-approved (STUDY00011346; STUDY202500751) project will comprise three phases: Program Development (year one), Provider Training (years one and two), and Testing the Self-Management Platform (year two). As per our HELP PAIN project, the Consolidated Framework for Implementation Research (CFIR) will be used to identify potential barriers and facilitators of implementation [29,38].

#### 2.1.1. Phase 1: Program Development

Our team of interdisciplinary academic researchers and community partners will create the PURSUIT provider training program (described below) and the companion online self-management platform for youth and families.

#### 2.1.2. Phase 2: PURSUIT Provider Training

The PURSUIT provider training program will be delivered in coordination with MASN and our state-level partner. Our team has longstanding relationships with MASN and our state level partner, and this project reflects an expansion of these partnerships [29,34]. We will use a blend of live and asynchronous training activities to reach the maximum number of providers, collecting feasibility, acceptability, and knowledge data immediately before and after training, as well as adoption data monthly for three months following training.

#### 2.1.3. Phase 3: Testing the Self-Management Platform

We will enroll Michigan students in elementary, middle, or high school and one caregiver to engage with the self-management platform. Measures of feasibility/acceptability, youth and caregiver’s physical and mental health symptoms will be collected pre- and post-engagement, as detailed below.

### 2.2. Eligibility Criteria and Projected Sample Sizes

#### 2.2.1. Provider Training

Any physical or mental healthcare professional will be eligible for enrollment. Due to our partnerships with organizations that employ and/or engage individuals serving schools, we expect most providers will be school-based professionals. We also expect most to be nurses, physicians, social workers, or addiction professionals based on the availability of continuing education (CE) credits (see Phase 1.3, below). However, neither school-based practice nor certification as a nurse, physician, social worker or addiction professional is required for enrollment. We aim to recruit 200 providers based on attendance at HELP PAIN training events [34,39].

#### 2.2.2. Youth/Caregivers

Youth aged 8 to 18 years enrolled in a Michigan elementary, middle, or high school may enroll with their legal caregiver to test the self-management platform. Access to a PURSUIT-trained provider is not a requirement for enrollment but will be assessed. As the current project is implementation-focused, enrollment criteria are more inclusive compared to traditional behavioral trials. We aim to recruit 100 individual youth/caregiver dyads based on our prior work.

### 2.3. Study Procedures

#### 2.3.1. Phase 1.1: Program Development

##### Development Team

Similarly to our earlier work [29], the PURSUIT provider training program and youth/caregiver self-management platform will be co-developed by an interdisciplinary team of academic and community partners. Academic researchers with expertise in pediatric psychology (NRC) and pediatric nursing (TSC) will create the initial materials (example provider training modules outlined in Table 2). The content will be reviewed by other experts in pediatric mental health, nursing science, pediatric pain, trauma, and substance use. Specifically, our team includes a pediatric clinical psychologist with expertise in chronic pain, trauma exposure, and stress (SN), a clinical psychologist focused on child mental health access and population-level preventative behavioral interventions (CC), a PhD-prepared psychiatric/mental health nurse practitioner whose work develops and tests technology-based health-promotion and mental health interventions (AC-CC), a community psychologist, clinical social worker, and community-engaged substance use researcher (MRR), a methodologist (SJP), and an addiction medicine physician/advocate (CP) who founded the Michigan Collaborative Addiction Resources and Education System (MI CARES; https://micaresed.org/ Accessed on 30 October 2025), the largest addiction medicine workforce development training program for prescribers and therapists in the nation. Our state partners will be represented by an advanced practice provider and clinical social worker with leadership roles in school health. Finally, our team will also include a district nurse and former HELP PAIN trainee (KT).

##### Training Content

In response to feedback from HELP PAIN provider-trainees and our funders, the PURSUIT training program will include content across four areas: (1) core cognitive–behavioral and mindfulness skills broadly applicable to pediatric physical and mental- health concerns, (2) pain management, (3) trauma-focused care, and (4) substance use prevention strategies for those aged 12 and older. This will enable different types of providers (e.g., nurses, mental health professionals) with varying levels of expertise to engage with the content most relevant and appropriate for their practice.

The PURSUIT core skills will apply to a variety of symptoms, including anxiety, low mood, stress, and fatigue. These may include psychoeducation (identifying connections among thoughts, feelings, behaviors, and symptoms), cognitive (identifying/challenging unhelpful thoughts), behavioral (relaxation skills, pleasant activity scheduling), and mindfulness (mindful breathing, mindful eating) coping strategies (see Table 2). Pain management strategies will be derived from HELP PAIN [29,34], and will include pain education, caregiver guidelines, and activity pacing [29,40,41]. The trauma-focused care content will focus on recognizing and responding to trauma symptoms in the school setting, and draw from evidence-based cognitive–behavioral protocols [42] to provide an introduction to developing a trauma narrative (educational in nature for non-mental health specialists). Finally, substance use prevention materials for youth aged 12 and older such as screening and motivational interviewing will be informed by content from the MI CARES Adolescents and Young Adults modules, as well as the American Academy of Pediatrics [43].

##### Innovative Training Aids

Our team will develop engaging training modules adaptable to live and asynchronous (i.e., recorded) delivery across these areas. All modules will incorporate visual aids (e.g., figures, videos), reflective questions, and case-based exercises to optimize participant engagement [44]. The team will script example patient-provider interactions to be recorded with live patient-actors and create videos explaining the foundational concepts for each content area, including possible adaptations of skills to different developmental levels and populations (e.g., neurodivergent youth). We will also develop a Digital Storytelling (DST) learning tool. DST is an approach to personal narrative synthesis that blends oral and image-based storytelling to promote healthy behaviors [45,46,47]. Finally, we will include animated videos of a youth and provider using pain management skills, which were part of a Swedish adaptation of a cognitive–behavioral program for pediatric functional abdominal pain and anxiety created by the senior author [40,41,48]. All videos will be embedded in the training modules and will be freely available online [https://www.youtube.com/@MSUhelplab Accessed on 30 October 2025]. Finally, to support providers’ use of PURSUIT skills in practice, we will create and distribute a comprehensive toolkit of screening measures, handouts, and worksheets.

##### Billing Content

Importantly, we will create training content situating PURSUIT skills within billing processes already used by school professionals. This content was requested by our funder, the Michigan Health Endowment Fund, as a way to support PURSUIT’s adoption and sustainability. Specifically, we will establish how delivery of PURSUIT strategies may count as a reimbursable service for providers using the Medicaid cost-based reimbursement model, if relevant to a child’s care plan and within scope of practice. We will also provide a list of potentially appropriate billing codes and modifiers for nurses and mental health professionals.

##### Self-Management Portal

In addition to the provider training materials, we will develop an online self-management platform for youth and caregivers. The self-management platform will provide education and coping skills to support youth and caregiver functioning and is not intended to replace physical or behavioral healthcare. We will, however, include contact information for national and state-level crisis supports within the platform out of an abundance of caution. The platform will be hosted on the Computerized Intervention Authorizing System (CIAS) 3.0, a virtual forum that can be accessed from most devices, utilizes features such as interactive avatars to engage users into skill building, and supports full HIPAA compliance. Unlike the provider training materials, content in the self-management portal will be presented as a cohesive program rather than as separate modules. However, the substance use prevention content will be available only for those aged 12 and older. The self-management materials will include the animated and live skill demonstration videos developed during the PURSUIT provider training program, as well as worksheets, handouts, and guidelines for caregivers of children with chronic pain.

#### 2.3.2. Phase 1.2 Iterative Refinement

The PURSUIT provider training content and companion self-management platform will be shared with representatives from our community and state partners, who will offer critical review and feedback as clinician-leaders. We will make subsequent modifications based on their recommendations, iteratively reviewing and refining the materials until a final product is achieved.

#### 2.3.3. Phase 1.3 Continuing Education Credit Processes

Ensuring that PURSUIT trainees receive continuing education credit (CE) is critically important to the research team, community partners, and funder as a means of promoting provider uptake. This process will entail partnering with accredited CE providers. Each module will be assigned credits based on length (see projections, Table 2).

To streamline coordination across specialties, we will utilize “self-claim” processes, allowing different types of providers (e.g., nurses, physicians, social workers) to claim credit for attending the same event. This will require working closely with representation from the credit-granting bodies to ensure that the live and asynchronous PURSUIT training activities meet specifications of each.

#### 2.3.4. Phase 2. Provider Training

We will work with representatives from our community and state partners to identify opportunities for live and/or asynchronous delivery of the PURSUIT training program. To promote attendance, we will strategize opportunities to deliver all four modules together over the course of a full day, as well as deliveries of single modules. Providers can elect to attend as many of the single-module deliveries as desired. We will be positioned to flexibly conduct these activities in person or over videoconferencing. Training sessions will be conducted by the lead investigator/senior author (a clinical psychologist), or a postdoctoral nurse practitioner and an advanced doctoral student in pediatric clinical psychology, both of whom will be trained by the lead investigator.

Our asynchronous recorded modules (recorded by the team members described above) will be freely available online, allowing for individually paced participation. Importantly, providers have the option of being trained in PURSUIT skills with or without participating in our research (see Provider Measures), further increasing programmatic reach.

#### 2.3.5. Phase 3. Child Outcomes

In the final phase of the project, we will make the self-management platform available to Michigan youth aged 8 through 18 enrolled in elementary, middle, or high school and one caregiver. Potential participants will be recruited using a flyer/email blast distributed at schools staffed by community/state partner-affiliated school providers, as well as through social media postings.

### 2.4. Assessment and Outcome Measures

#### 2.4.1. Provider Measures

We will collect data from providers immediately before, after, and monthly for three months following PURSUIT training. As providers may selectively choose which training modules to engage with, some data (e.g., knowledge, feasibility and acceptability, anticipated use) are collected for every module completed, while others (e.g., school climate) are reported once per provider (see Table 3). However, we note variations based on whether providers participate in a live all-day training where modules are delivered together, or single-module deliveries (Table 3).

#### 2.4.2. Feasibility and Acceptability

The feasibility of the provider training program will be assessed using completion rates (i.e., the percent of providers who complete a post-assessment measure, among those who begin at least one training module). We will also explore the average number of training modules completed per provider.

Providers will respond to a 12-item measure of perceived feasibility (“PURSUIT seems implementable”), acceptability (“PURSUIT meets my approval”), and appropriateness (“PURSUIT seems applicable”) for each module completed, or once if attending an all-day training (i.e., all modules delivered together over the course of a day) [33,34]. Items are grouped into one of three four-item subscales (feasibility, acceptability, or appropriateness of the program) and rated on a 5-point Likert-type scale (1 = completely disagree, 5 = completely agree); responses are summed and averaged to yield separate subscale scores [33]. Prior research has established the internal consistency (Cronbach α = 0.89 feasibility, 0.85 acceptability, 0.91 appropriateness), construct validity, and test–retest reliability (Pearson’s *r* = 0.88 feasibility, 0.80 acceptability, 0.73 appropriateness) of the subscales [33].

Finally, providers will complete a semi-structured interview or commensurate open-ended survey questions (i.e., “Did you like the way that [skill] was presented? What did you like/not like?”), as per our prior work [29,49]. These will be completed as open-ended survey questions for asynchronous trainings, or as semi-structured interviews for live in-person and virtual trainings. Interviews will be audio-recorded and transcribed for thematic analyses. Providers will offer feedback for the program overall if attending an all-day training or for each module separately if completing single-module deliveries.

#### 2.4.3. Provider Knowledge

Providers will complete a 10-item (core skills) or 5-item (pain management, trauma-focused care, substance use prevention) multiple-choice quiz assessing their knowledge of symptom management skills before and after each training module, as per our prior work [34]. Each item is scored 0 = incorrect or 1 = correct and summed.

#### 2.4.4. Anticipated Use

Providers will report the estimated proportion of youth they typically work with within a year who may benefit from PURSUIT skills following the completion of each module, or once at the end of an all-day training. They will also report on the presence (yes/no) of 13 potential barriers (i.e., insufficient time or space) and 13 potential facilitators (i.e., school staff or parent support, youth interest) to using the skills with youth in their setting. Endorsed barriers/facilitators will be separately summed (range 0–13).

#### 2.4.5. School Climate

Providers will respond to a measure of school climate before completing their first PURSUIT training module, adapted from the school personnel version of the freely available School Climate School Survey Suite [50]. The original 29-item measure assesses school climate across the subscales of staff connectedness, learning and physical environments, school safety, and student/adult relationships. Items are scored on a 4-point Likert scale (0 = strongly agree, 4 = strongly disagree) and summed to yield an overall and separate subscale scores [50,51]. The internal reliability (Cronbach α = 0.95 overall; 0.78–0.95 per subscale), construct, and convergent validity have been established [50,51]. Our adaptations include adding eight items probing the availability of resources to address student health concerns, teacher/staff and parental support for school-based healthcare services, and students’ level of ease accessing healthcare services in the school.

#### 2.4.6. Monthly Use and Adaptation

For three months following completion of their first PURSUIT training module or an all-day training, providers will report how many times they used a PURSUIT skill with a youth in the past month, the specific skill(s) used, and—if applicable—the reasons they have not used PURSUIT with youth who may benefit. Providers will also report whether they have used PURSUIT skills with youth who have a co-occurring physical, psychological, or neurodevelopmental condition, and whether/how they adapted the skill for that child. Finally, providers will report the number of PURSUIT-related Medicaid billing claims submitted, and if they have trained any other care professionals to use PURSUIT skills.

#### 2.4.7. Demographics

Before completing their first PURSUIT module, providers will report their demographics (age, gender identity, race, and ethnicity), as well as their terminal degree, years of experience, and practice setting (e.g., number and type of schools served).

#### 2.4.8. Child Measures

All youth reported measures will be collected at baseline and post-assessment, approximately eight weeks later (see Table 3).

#### 2.4.9. Feasibility and Acceptability

The feasibility and acceptability of the online self-management platform will be assessed using retention (i.e., the percentage of youth who complete a post-assessment measure, among those who complete a baseline assessment). We will also assess the percentage of youth who engage with the platform (i.e., complete at least one activity) among those who complete a baseline assessment, and the average number of completed activities. Finally, we will conduct semi-structured interviews with youth following the engagement period to assess feasibility (“Do you think you will be able to integrate these skills into your schedule with school/work?”) and acceptability (“What are some of the benefits of using PURSUIT? Drawbacks?”) [49].

#### 2.4.10. Contact with a PURSUIT Provider

While not a requirement for enrollment, youth will report whether they are working with a PURSUIT-trained school provider, the frequency of their work together, and which skills have been introduced to them in that setting.

#### 2.4.11. Pain

Youth will report their average pain intensity over the past two weeks using a single-item Visual Analog Scale (VAS), which has been validated for children aged 8 and older [52,53]. Responses range from 0 (no pain) to 10 (worst imaginable pain), with ratings  ≥  3 indicating moderate pain [54].

#### 2.4.12. Functional Disability

Youths’ difficulty performing tasks across home, school, recreational, and social settings over the “past few days” due to physical symptoms will be reported by youth using the valid and reliable 15-item Functional Disability Inventory (FDI; Cronbach’s α = 0.86–0.91; two week test–retest reliability *r* = 0.74; appropriate for use among youth aged 8 to 18 years) [55,56,57]. Items such as “being awake all day without a nap/rest” are rated from 0 (“no trouble”) to 4 (“impossible”) and summed, yielding total scores of 0–60 with scores ≥ 7 suggesting greater than minimal disability [55].

#### 2.4.13. Post Traumatic Stress Disorder (PTSD) Symptoms

Youth who endorse prior exposure to a frightening or upsetting event (yes/no; details not queried) will complete the UCLA Reaction Index for DSM-5 Brief Screening Form, an 11-item measure of how often they experienced psychological stress and physiological reactivity due to internal or external trauma reminders, intrusive trauma-related memories, avoidance of internal and external reminders of trauma, emotional dysregulation, attention issues, feelings of detachment, and sleep disturbance over the past month [58]. Items are scored from zero (none of the days) to four (most of the days) and summed; scores of 11–20 suggest mild PTSD symptoms, while scores ≥21 are indicative of potential PTSD. Prior work has established the criterion validity and diagnostic accuracy of this measure for youth aged 7 to 18 years [59].

#### 2.4.14. Anxiety

Youths’ past three-month anxiety symptoms will be assessed using the 41-item Screen for Child Anxiety-Related Disorders (SCARED) [60]. Items are scored on a 3-point Likert-style scale (0  =  not true or hardly ever true, 2 = very true or often true), and summed to yield total scores of 0–82 with higher scores indicating greater anxiety and scores ≥ 25 suggesting clinical significance [61,62]. The SCARED has been used among youth age eight to 18 years, with established internal reliability, test–retest reliability, and discriminant validity (Cronbach’s α = 0.92; Pearson’s *r* = 0.78) [62,63,64].

#### 2.4.15. Depressive Symptoms

Youth will report the frequency (1 = never, 5 = almost always) of past-week depressive symptoms (e.g., sadness, loneliness) using the eight-item PROMIS Pediatric Depressive Symptoms Short Form 8a [65]. Raw summed scores are converted to T-scores (mean 50, standard deviation 10), with T-scores >55 suggesting mild or higher symptoms. Content validity and reliability have been established for this measure (Cronbach’s α = 0.85–0.86; ICC = 0.76) [66], which is appropriate for use among youth aged 8 to 18 years [67].

#### 2.4.16. Stress

Youth will report how often (1 = never, 5 = almost always) they experienced feelings of stress over the past week using the eight-item PROMIS Psychological Stress Experiences Short Form 8a [68,69]. As per other PROMIS short forms, summed scores are converted to T-scores. The construct validity and reliability (Cronbach’s α = 0.92; ICC = 0.71) of this measure (appropriate for ages 8 to 18 years) have been established [68].

#### 2.4.17. Fatigue

Past week fatigue symptoms will be self-reported by youth using the 10-item PROMIS Pediatric Short Form (appropriate for ages 8 to 18 years) [67,70]. Items (“Being tired made it hard for me to play or go out with my friends as much as I’d like”) are rated on a 5-point Likert-style scale and summed, then converted into T-scores. The measure’s convergent/discriminant validity and reliability have been demonstrated (Cronbach’s α = 0.87; ICC = 0.76) [66].

#### 2.4.18. Sleep Disturbance

The frequency (1 = not at all, 5 = very much) with which youth experienced sleep disturbance symptoms in the past week will be assessed using the eight-item PROMIS Pediatric Sleep Disturbance Short Form (appropriate for youth aged 8 to 18 years) [71,72]. T-scores will be derived from summed raw scores. Internal reliability and convergent validity have been established for this measure (Cronbach’s α = 0.97) [71].

#### 2.4.19. Perceived Risk of Substance Use

Youth age 12 and older will report how risky (1 = no risk, 4 = great risk) they perceive different cigarette, cannabis, alcohol, or vaping use behaviors (e.g., occasional versus regular use) using 12 items from the 2023 Monitoring the Future (MTF) study [73]. MTF is an ongoing longitudinal panel study conducted by the University of Michigan Institute for Social Research that includes annual surveys of nationally representative samples of 8th, 10th, and 12th grade youth.

#### 2.4.20. Substance Use Behaviors

The frequency (never, once or twice, monthly, weekly or more often) of past-year nicotine, alcohol, cannabis, and other substance use behaviors will be assessed for youth 12 years and older using the youth-reported Screening to Brief Intervention (S2BI), a seven-item, validated measure with established clinical utility [74,75].

#### 2.4.21. Patient Global Functioning and Impression of Change

Youth and caregivers will separately report on the child’s overall or global functioning (i.e., how much symptoms disrupt everyday life) on a 0–100 scale before and after engaging with PURSUIT, with higher scores suggesting better functioning [76]. Following engagement, youth and caregivers will also complete a single-item measure of perceived change in the child’s overall functioning on a scale of 1 (very much improved) to 7 (very much worse), with 4 (no change) as the midpoint [77]. This measure is widely used in pediatric clinical pain research; its clinical utility and responsiveness to change are established [77,78].

### 2.5. Caregiver Measures

#### 2.5.1. Adverse Childhood Experiences (ACEs)

Youth’s lifetime exposure to nine ACEs (e.g., violence, household dysfunction) will be reported by caregivers at baseline (if youth age < 18) using items adapted from the National Survey of Children’s Health [79]. Endorsed exposures are scored as 1, with summed scores ranging from 0 to 9 [79].

#### 2.5.2. Caregiver Wellbeing

Caregivers will self-report how often they experienced symptoms of depression, anxiety, and stress in the last week using the 21-item Depression Anxiety Inventory Stress Scales (DASS21) at baseline and post-assessment [80]. The items are scored on a 4-point Likert-style scale and summed into three separate depression, anxiety, and stress subscales, each with established cut points indicating level of severity (e.g., normal, mild, moderate, severe, extremely severe). The internal reliability (Cronbach’s α = 0.89–0.93), convergent, and discriminant validity of the DASS21 have been established [81].

#### 2.5.3. Demographics

Caregivers will report youths’ age, sex at birth, gender, racial, and ethnic identities, household income, health insurance status/type, and their own highest level of education at baseline. They will also report the frequency with which their child misses, leaves early, or arrives late to school due to pain, mental health symptoms, or other illnesses over the past month and past week at baseline and after engaging with the self-management platform. These data will be self-reported by youth aged 18.

## 3. Analytic Plan

### 3.1. Provider Outcomes

#### 3.1.1. Quantitative Data

We will conduct initial data analyses that describe the amounts and patterns of missing data, univariate distributions, and multivariate associations between variables (e.g., frequencies, cross-tabulations, chi-squared or *t*-tests depending on variable type, data visualization). Next, we will turn to analyses that test the substantive hypotheses [82,83,84]. The hypotheses for our primary outcomes test whether the study attained benchmark levels of feasibility, acceptability, and appropriateness. The non-inferiority testing framework for doing that requires setting a margin that represents trivial deviation from the benchmark [85,86]. Few papers that employ equivalence or non-inferiority tests justify the margins used, with meta-analyses of medical studies suggesting only 20% to 46% of studies do so [85,87,88].

One-sided Wilson score 95% confidence intervals for proportions with continuity correction will be used to conduct non-inferiority tests on outcomes with benchmarks defined as percentages [89,90]. We will examine whether the percentage of providers who complete a post-assessment measure for at least one initiated training module is not inferior to the 75% benchmark [86,91], within a margin of 5.7% (*H1A*). Using a smaller margin for this hypothesis would make it empirically impossible to detect non-inferiority unless the observed rate is substantially higher than the benchmark or the sample size exceeds the recruitment goal because the width of a proportion’s confidence interval is determined by both the proportion itself and sample size. For context, margins of 5% have been used to compare the prevalence of health conditions detected between population-based surveys and electronic health records and to compare surgery revision, complication, and re-admission rates [92,93]. Given the sample size, benchmark, margin, and confidence level, this test should have 80% power to detect noninferiority if the true provider completion rate is ≥77.3%.

The average number of training modules completed per provider will be computed as a measure of training dosage. We will use non-inferiority tests [86] to examine whether providers’ mean responses to the three feasibility, acceptability, and appropriateness subscales are not inferior to the benchmark (4, “agree”), within a margin of 0.2 points (5%) (*H1B*). Detecting non-inferiority with this margin requires that the lower 95% confidence limit for the mean exceed 3.8, a value much closer to the benchmark than to the neutral anchor (3, “neither agree nor disagree”) for the items measuring these subscales. Given the sample size, benchmark, margin, and confidence level, these tests should have ≥ 81% power to detect noninferiority if the true means are ≥ 3.98.

Changes in providers’ knowledge of management skills from pre- to post-training (*H1C*) will be examined using univariate latent change score (LCS) modeling, a form of structural equation modeling which conceptualizes scores as the function of an observed autoregressive component and an unobserved latent change factor [94,95,96]. Specifically, we will estimate separate models for each training module (core skills, pain management, trauma-focused care, substance use prevention) using pre-training quiz scores as the autoregressive predictors of post-training quiz scores, with the latent change factor defined by fixing the regression weight of post-training scores on pre-training scores to equal one [94,96]. The mean of the latent change factor will reflect average change in knowledge from pre- to post-training (i.e., average within provider change), while the variance will quantify variation in individual change trajectories (i.e., between-provider change) [96]. Regressing latent change on to pre-training scores will allow us to examine the extent to which change is dependent upon pre-training knowledge levels (i.e., proportional change) [94]. We will compare these models to models where the latent change factor mean is constrained to zero using a chi-squared difference test, to test the hypothesis that there is no change in provider knowledge [94]. For training modalities (i.e., live in-person, live virtual, or asynchronous) with sufficient numbers of participants, differences in knowledge change across modality will be examined using multi-group LCS modeling. Chi-square difference tests will assess whether setting equality constraints on the latent change mean, variance, and proportional change parameters affects model fit [94]. We will assess whether it is feasible and necessary to account for provider clustering within training events in the LCS models, switching to multilevel LCS models if so.

Finally, we will estimate the percentage of providers using PURSUIT skills on a monthly basis with corresponding two-sided 95% Wilson score confidence intervals and use descriptive statistics to characterize the number of youth with whom providers report using PURSUIT skills on a monthly basis, the average number of skills used per youth, and PURSUIT-related Medicaid billing submissions.

#### 3.1.2. Qualitative Data

We will use grounded theory [97] and thematic analytic approaches [98] to derive common themes from provider responses to a brief, semi-structured interview or open-ended survey questions (*H1B*). In line with our earlier work [41,99], two or more members of the study team will iteratively code, categorize, and theme the transcribed interviews and survey responses until data saturation is achieved [100]. After coding is complete, the team will develop categories and themes. The reviewers will meet regularly to discuss emerging themes, and discrepancies will be resolved via discussion and revisiting the data for additional context until consensus is achieved [92].

### 3.2. Youth Outcomes

#### 3.2.1. Quantitative Data

Following initial data analyses similar to those employed for provider data, we will assess the feasibility and acceptability of the PURSUIT self-management platform using non-inferiority tests to examine whether (a) the retention rate is not inferior to the 75% benchmark within an 8.3% margin (*H3A*) and (b) the percentage of youth who engage with the content (i.e., complete at least one activity) is not inferior to the 70% benchmark with an 8.6% margin (*H3B*). These are the smallest margins that could be empirically met given expected sampling error if the estimated rates match the corresponding benchmark rates and sample size reaches the recruitment goal. Given the sample size, specified benchmarks and margins, and 95% confidence levels, these tests should have ≥80% power to detect noninferiority if the true youth retention and engagement rates are respectively ≥78.0% and ≥73.3%. We will also describe the average number of activities youth complete within the platform over the engagement period as a dosage measure.

Changes in youth and parent symptomatology (e.g., anxiety, depression, stress, mood, pain) from baseline will be estimated using LCS modeling as described above (*H3C*, *H3D*). In particular, we will compare models where mean change is unconstrained versus constrained to zero to test the hypothesis that there was no change in symptomatology. We will assess whether it is feasible and necessary to account for youth or caregiver clustering within schools in the LCS models, switching to multilevel LCS models if so.

#### 3.2.2. Qualitative Data

We will analyze youth’s responses to open-ended questions or a semi-structured interview using the processes described above for provider data.

### 3.3. Multiple Testing

Adjustments for multiple testing aim to control the familywise error rate across the set of hypotheses tested as part of a single experiment or family [101]. They require considering which hypotheses should be grouped together. This study prioritizes feasibility, acceptability, and appropriateness as primary outcomes separately for providers (*H1A* and *H1B*) and youth (*H3A* and *H3B*). Using distinct samples of participants who interact with the program in different ways is like having two different experiments [101]. Because the number of primary outcomes for each sample is very small, we will not adjust for multiple testing on these analyses.

Provider knowledge change (*H1C*) and improvement in youth and caregiver symptoms (*H3C* and *H3D*) are measures of program effectiveness. They are secondary outcomes and the subjects of exploratory analyses focused on obtaining preliminary effect size estimates and confidence intervals, which does not warrant multiplicity adjustment [102].

### 3.4. Minimum Detectable Effect Size for Latent Change Score Models

The simplest forms of LCS models are functionally equivalent to paired *t*-tests [103]. Therefore, power analysis methods for paired *t*-tests were used to discern the minimum detectable effect size (MDES, measured by Cohen’s *d*) for testing whether mean longitudinal change scores differ from zero (*H1C*, *H3C*, and *H3D*). At the planned sample sizes, and α = 0.05, the MDES at 80% power is *d* = 0.20 for provider outcomes and *d* = 0.28 for youth/caregiver outcomes. Even at α = 0.01, the MDES values at 80% power remain small: *d* = 0.24 and 0.35, respectively. The study should therefore be able to detect small longitudinal changes in provider knowledge and youth or caregiver symptoms.

### 3.5. Handling Missing Data

The rate and patterns of missing data will inform how they are handled during quantitative analyses. Multiple imputation will be used when necessary to preserve power or reduce bias associated with complete case analysis.

## 4. Discussion

This protocol paper describes our planned approach to developing, implementing, and evaluating PURSUIT, a training program for school providers managing common youth physical and mental health symptoms and a companion online self-management platform for youth and caregivers. If supported by future evidence, our work may inform school-based practice in Michigan and beyond. Indeed, by integrating continuing education credit and billing relevance, we hope to develop a program with the potential for long-term sustainability and scalability.

PURSUIT was conceptualized in direct response to requests from provider trainees for additional physical and mental health content (including trauma-related care and substance use prevention strategies) following HELP PAIN implementation. These requests reflect what providers see in their daily practice and may not have come to our attention without having established open lines of communication with community partners. This critical feedback was elicited in the context of a relationship built over time and grounded in repeated demonstrations of our teams’ genuine investment and partnership with trusted entities like MASN [29,34]. Furthermore, our work has benefitted from the application of a cultural adaptation model, which provides a framework for effectively partnering with local teams as a means for moving research into practice [38].

PURSUIT will be developed as an introductory support program for school providers, youth, and caregivers managing common physical and mental health challenges including stress, anxiety, mood problems, pain, trauma-related symptoms, and risk for substance use. Notably, youth with complex and/or severe symptoms or risk factors may require an escalated level of care, including emergency intervention. We will include information in our training program to support providers making that determination, including screening/assessment tools, conversation guides, and resources for finding treatment options in their area. The companion self-management platform will clarify that PURSUIT was designed as a support tool for education and coping skill-development and is not intended to replace medical and/or behavioral healthcare provided inside or outside of the school setting.

Limitations to our planned protocol for co-development include a lack of partnership with organizations in other areas of Michigan (e.g., Detroit, Flint) and representation from Indigenous communities, which have experienced historical and ongoing traumas that may shape members’ need for care. However, both our community (MASN) and state partners have state-wide reach, suggesting that providers from these communities may engage in PURSUIT training. As we iteratively co-develop and refine the PURSUIT program, we may identify opportunities to address this gap and develop guidance on how providers can incorporate cultural humility into the framework for delivery [104] and tailor materials to best serve their communities.

## 5. Conclusions

Our interdisciplinary team spanning nursing, psychology, social work, and medicine, and interinstitutional partners across community, professional, and governmental organizations conceptualized and will co-develop the PURSUIT program in direct response to provider- and funder-identified needs. As per our earlier work, we will leverage community partnerships to optimize programmatic relevancy and dissemination. These partnerships rely on mutual trust and an uninhibited bidirectional exchange of information. We believe that PURSUIT may have the potential to expand access to high-quality care for youth and may positively impact physical and mental health outcomes.

## Figures and Tables

**Table 1:** RE-AIM framework.

Dimension	Target	Aim/Hypotheses
Reach	Number and percent of providers and youth retained.	Aim 1: H1A; Aim 3: H3A
Effectiveness	Increased provider knowledge; reduced youth/caregiver symptoms.	Aim 1: H1C; Aim 3: H3C, H3D
Adoption	Providers’ monthly use of PURSUIT skills; the percent of youth who engage with the online self-management platform.	Aim 2; Aim 3: H3B
Implementation	Feasibility, acceptability, knowledge.	Aim 1: H1A-B; Aim 3: H3A-B
Maintenance	Providers’ monthly use of PURSUIT skills and frequency of PURSUIT-related billing submissions; percentage of providers who report training other professionals in skills.	Aim 2

**Table 2:** Example PURSUIT training modules.

Core Skills	Pain Management	Trauma Focused Care	Substance Use Prevention
Psychoeducation - The link between thoughts, feelings, and actions	Pain education - Gate control theory - Guidelines for caregivers	Trauma basics - Screening for trauma - Responding to trauma symptoms in schools	Adolescent substance use trends
Relaxation training - Deep breathing - Mindful breathing - Progressive muscle relaxation - Guided imagery	- Screening for pain and pain-related functional disability in schools	Identifying emotions	Screening practices
Cognitive restructuring - Challenging unhelpful thoughts - Detective thinking - Problem solving and future planning	Pain coping skills - Core skill adaptations - Activity pacing - Challenging negative thoughts	Coping skills for youth experiencing trauma symptoms - Core skill adaptations - Grounding skills	Addressing perceived risk and preventing substance use - Motivational interviewing - Assertiveness skills adaptations
Other behavioral skills - Mindful eating - Pleasant activity scheduling - Fighting fears by facing fears (exposure)	Developing appropriate school accommodations	Creating a trauma narrative	
Health promotion - Appropriate self-assertion - Sleep hygiene		Graduated exposure	
Projected continuing education (CE) credits per module: 3	Projected CE credits: 2	Projected CE credits: 2	Projected CE credits: 1

**Table 3:** Measures and collection plan ^1,2^.

**Provider Measures**			
**Measure**	**Pre-Training**	**Post-Training**	**Monthly for 3 Months ^3^**
Demographics	•		
Feasibility, Acceptability, Appropriateness Subscales *Per module completed; once if all-day training*		•	
Qualitative interview or open-ended survey *Per module completed; once if all-day training*		•	
Knowledge quiz *Per module completed*	•	•	
Anticipated use of PURSUIT *Per module completed; once if all-day training*		•	
School climate *Once per participant*	•		
Monthly use of PURSUIT skills with youth			•
Adaptations for youth with co-occurring conditions			•
PURSUIT-related billing submissions			•
Training other professionals in PURSUIT			•
**Youth Measures**			
**Measure**	**Pre-engagement**	**Post-engagement**	
Contact with a PURSUIT Provider	•	•	
Pain Intensity Visual Analog Scale (VAS)	•	•	
Functional Disability (FDI)	•	•	
Post-traumatic Stress Disorder Symptoms (UCLA Reaction Index for DSM-5 Brief Screening Form) ^4^	•	•	
Anxiety (SCARED)	•	•	
Depressive Symptoms (PROMIS Short Form)	•	•	
Stress (PROMIS Short Form)	•	•	
Fatigue (PROMIS Short Form)	•	•	
Sleep Disturbance (PROMIS Short Form)	•	•	
Perceived Risk of Substance Use (Monitoring the Future items)	•	•	
Substance Use Behaviors (S2BI)	•	•	
Patient Global Functioning and Impression of Change	•	•	
Qualitative Interview		•	
**Caregiver Measures**			
**Measure**	**Pre-engagement**	**Post-engagement**	
Demographics	•		
Patient Global Functioning and Impression of Change	•	•	
Youth Adverse Childhood Experiences (ACE) Exposure	•		
Depression Anxiety Inventory Stress Scale (DASS21)	•	•	

^1^ Data collection and management processes have been reviewed and approved by the Michigan State University Institutional Review Board, in full compliance with university standards for the protection of human subjects. ^2^ We aim to collect data from 200 providers and 100 youth/caregiver dyads, based on our prior related work. ^3^ Monthly from the completion of the first PURSUIT module or an all-day training. ^4^ Completed by youth who endorse exposure to a frightening or upsetting event.

## Data Availability

No data were created.

## Abbreviations

The following abbreviations are used in this manuscript:

U.S.	United States of America
MASN	Michigan Association of School Nurses
DST	Digital storytelling
CIAS	Computerized Intervention Authoring System
MI CARES	Michigan Collaborative Addiction Resources and Education System
CE	Continuing education
SCARED	Screen for Child Anxiety-Related Disorders
